# The relationship between frailty and multimorbidity in Chinese older adults: the chain mediating effects of sleep quality and anxiety

**DOI:** 10.1186/s12877-025-06490-8

**Published:** 2025-10-27

**Authors:** Ze Ma, Liang Zhou, Guoxian Li, Hanqing Zhao, Mengtong Sun, Yu Wang, Jianing Li, Yujie Shi, Zexin Lou, Ziqing Sun, Qiang Han, Miao Jiang, Yueping Shen

**Affiliations:** 1 Department of Epidemiology and Biostatistics, School of Public Health, Medical College of Soochow University, 199 Renai Road, Suzhou City, Jiangsu Province 215123 China; 2 The Fourth Affiliated Hospital of Soochow University, Suzhou City, Jiangsu Province 215000 China; 3 Liyang Center for Disease Control and Prevention, 209 Yongding Road, Liyang City, Jiangsu Province 213300 China

**Keywords:** Frailty, Multimorbidity, Sleep quality, Anxiety, Mediation analysis

## Abstract

**Background:**

Previous studies have shown an association between frailty and multimorbidity, but the underlying mechanisms are still unclear. Therefore, this study aimed to investigate the potential chain mediating roles of sleep quality and anxiety in the relationship between frailty and multimorbidity.

**Methods:**

This cross-sectional study used data from the first follow-up of the Liyang cohort study on chronic diseases and risk factors monitoring in China (Liyang Study), which comprised 2874 participants aged ≥ 60 years from 17 health centres in Liyang City. Multimorbidity was defined based on 13 self-reported chronic conditions. A modified version of the frailty phenotype was used to assess frailty. Sleep quality and anxiety were assessed using the Pittsburgh Sleep Quality Index and the 7-item Generalized Anxiety Disorder Scale, respectively. Spearman correlation analysis was employed to examine the correlations between frailty, sleep quality, anxiety, and multimorbidity. A chain mediation analysis of sleep quality and anxiety on the relationship between frailty and multimorbidity was conducted using the SPSS PROCESS macro (Model 6).

**Results:**

In this study, significant correlations were observed between frailty, sleep quality, anxiety, and multimorbidity (*P* < 0.01). Frailty had a direct impact on multimorbidity (unstandardised coefficient [B] = 0.196, bootstrap 95% confidence interval [CI] = [0.146, 0.246]). The association was mediated by three important pathways. (1) Sleep quality contributes 26.53% of the total effect (B = 0.052, bootstrap 95% CI = [0.038, 0.068]). (2) Anxiety accounts for 20.92% of the total effect (B = 0.041, bootstrap 95% CI = [0.025, 0.060]). (3) Sleep quality and anxiety represent 5.10% of the total effect (B = 0.010, bootstrap 95% CI = [0.006, 0.015]). The overall mediation effect was 52.55%.

**Conclusions:**

Frailty was positively associated with multimorbidity, and sleep quality and anxiety were important mediators of the association between frailty and multimorbidity. Addressing frailty, enhancing sleep quality, and promoting a positive mood may help reduce multimorbidity and enhance the overall well-being of elderly individuals. This study could potentially contribute to the development of more effective strategies to prevent multimorbidity.

**Supplementary Information:**

The online version contains supplementary material available at 10.1186/s12877-025-06490-8.

## Background

With an aging population worldwide, multimorbidity has become more widespread. Multimorbidity is generally defined as the simultaneous presence of two or more chronic diseases in the same person [1]. Data from the China Health and Retirement Longitudinal Study (CHARLS) showed that 42.4% of individuals aged ≥ 50 years in China have experienced multimorbidity [2]. Multimorbidity imposes enormous financial pressure on patients, family members, and healthcare systems, as individuals with multimorbidity are hospitalised more frequently and for much longer periods [3]. In addition, multimorbidity is associated with a series of adverse health consequences, such as poor health-related quality of life and premature death [4, 5]. Consequently, a growing body of studies have focused on the risk factors for multimorbidity in older adults, including frailty [6, 7]. Frailty is a dynamic and multidimensional syndrome characterised by reduced reserves and diminished resistance to stressors [8]. The prevalence of frailty has been documented to increase with advancing age [9]. A previous study conducted in the United Kingdom found that frailty was strongly associated with multimorbidity [10]. Previous studies have proposed that inflammation may serve as a shared pathogenic mechanism that contributes to both frailty and multimorbidity [11]. However, research on how frailty affects multimorbidity through sleep quality and anxiety, particularly in older Chinese adults, is scarce.

As age increases, sleep disorders occur because of the deterioration of various physiological functions [12]. A recent systematic review concluded that poor sleep quality and sleep duration exceeding the currently recommended levels are always associated with multimorbidity, despite varying degrees of strength [13]. A nationally representative longitudinal study also suggests that short sleep duration is associated with an increased risk of multimorbidity in Chinese middle-aged and older adults [14]. Inflammatory markers may serve as connecting links in this association [15]. Research has demonstrated an association between increased frailty in older individuals and decreased sleep quality, potentially exacerbated by frailty, leading to disrupted sleep rhythms that affect sleep quality [16]. Therefore, sleep quality may mediate the relationship between frailty and multimorbidity. In addition to sleep quality, anxiety is also a significant risk factor for multimorbidity. A longitudinal study conducted in the United States revealed that individuals experiencing anxiety exhibited an accelerated rate of chronic disease development [17]. Furthermore, several prior studies have documented a significant positive association between frailty and anxiety [18, 19]. One possible explanation is that individuals with frailty may experience anxiety owing to disabilities caused by reduced physical function [19]. Thus, anxiety may play a mediating role in the relationship between frailty and multimorbidity. In addition, emerging evidence suggests that sleep disturbance may be a more influential predictor of anxiety [20]. Hence, we hypothesised that sleep quality and anxiety may consecutively mediate the association between frailty and multimorbidity.

**Fig. 1:**
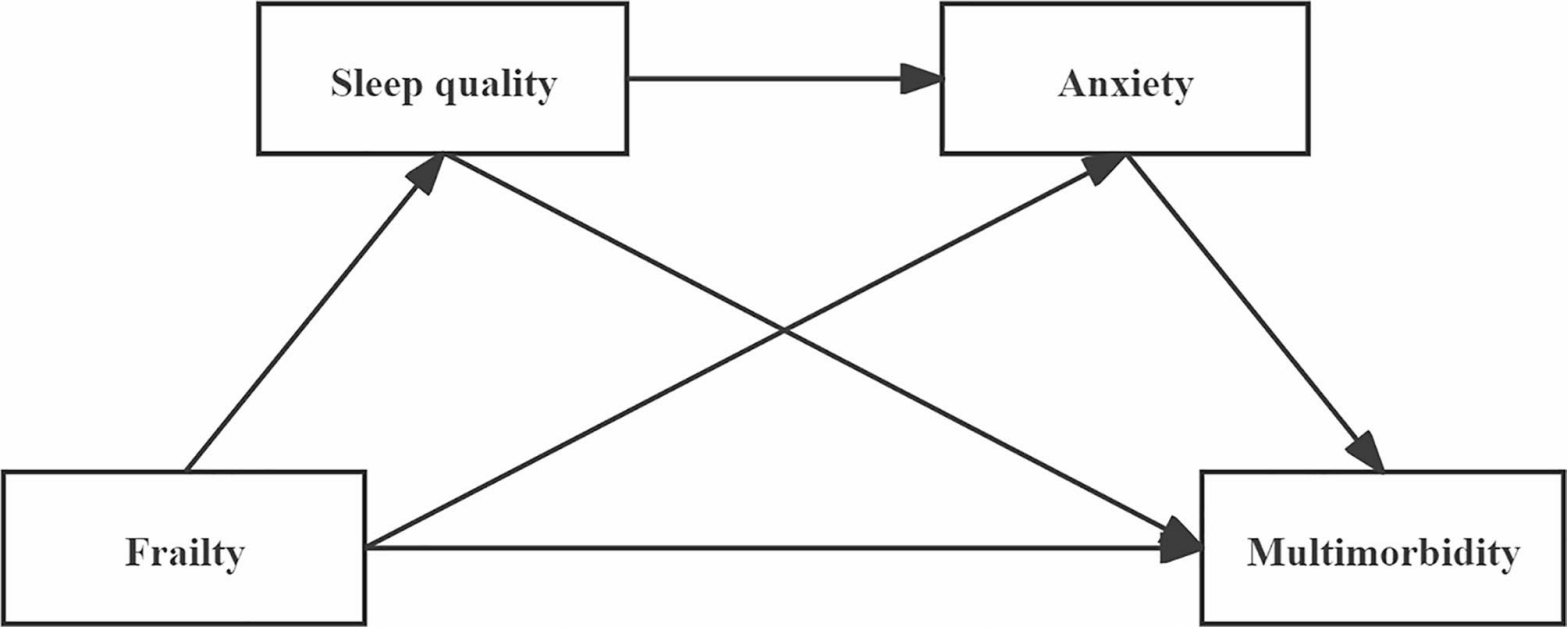
Hypothetical path model for this study

Given this background, the study aimed to investigate whether and how sleep quality and anxiety mediate the association between frailty and multimorbidity in elderly Chinese individuals. The following four hypotheses were proposed: (1) frailty positively predicts multimorbidity in older Chinese adults; (2) sleep quality mediates the association between frailty and multimorbidity; (3) anxiety mediates the relationship between frailty and multimorbidity; and (4) sleep quality and anxiety play a chain mediating role in the association between frailty and multimorbidity. The hypothetical path model used in this study is shown in Fig. 1.

## Methods

### Study design and participants

**Fig. 2:**
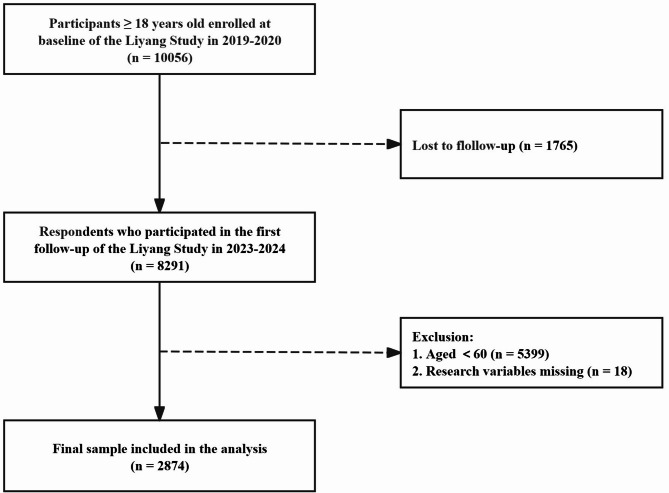
Flowchart of participant selection

The study used data from the first follow-up of the Liyang cohort study on chronic diseases and risk factors monitoring in China (Liyang Study). The prospective Liyang Study was initially intended to investigate the determinants of prevalent chronic non-communicable diseases. Further elaboration on the study design and data collection methods for the Liyang Study baseline survey can be found in the existing literature [21]. From March 2019 to June 2020, 10,056 participants aged ≥ 18 years were recruited using a multistage cluster random sampling method at 17 health centres in Liyang City, Jiangsu Province, China. The first follow-up of the Liyang Study was conducted from June 2023 to June 2024 and encompassed sociodemographic information, health-related behaviours, psychosocial factors, medical history, and other relevant data. Physicians from the 17 health facilities were trained to conduct in-person interviews and physical measurements using standardised questionnaires administered through computer-assisted personal interviews. Of the 10,056 individuals who participated in the baseline survey of the Liyang Study, 8291 participated in the first follow-up of the Liyang Study. The current study ultimately included 2874 participants aged ≥ 60 years with complete data on frailty, sleep quality, anxiety, multimorbidity, and covariates. An overview of the inclusion and exclusion criteria for this study population can be found in Fig. 2. The study was approved by the Ethics Committee of Soochow University (Approval No. SUDA20230915H01). Each participant was required to provide verbal informed consent before in-person interviews were conducted.

### Measures

#### Dependent variables

Multimorbidity was defined using 13 self-reported chronic conditions diagnosed by a physician, collected from the first follow-up of the Liyang Study. The original assessment protocol, aligned with the CHARLS, encompassed 14 clinically significant diseases prevalent in China’s aging population, including hypertension, memory-related disorders, dyslipidaemia, diabetes, kidney disease, cancer, chronic obstructive pulmonary disease, arthritis, liver disease, stomach problems, heart disease, stroke, emotional, nervous or psychiatric problems, and asthma [22]. After excluding emotional, nervous or psychiatric problems due to their conceptual overlap with anxiety (the mediator in these analyses), 13 diseases were retained [23, 24]. In our study, multimorbidity was measured as the summation of the number of chronic diseases and was expressed as a continuous variable to evaluate the severity of multimorbidity as well as the cumulative effect of chronic disease [25]. Each chronic condition was assigned a score of 1 if present and 0 if absent. The total multimorbidity score ranged from 0 to 13 points.

#### Independent variable

A modified version of the frailty phenotype created in the CHARLS, and validated for identifying frailty in older Chinese adults, was used to measure frailty [26, 27]. The method was widely used in previous works [28, 29]. The scale contained the following five items: exhaustion, inactivity, slowness, shrinking, and weakness (further details are provided in Supplementary Table 1). Each item meeting the specified criteria was assigned a score of 1 point, whereas those not meeting the criteria were assigned a score of 0 points. The total score ranged from 0 to 5, with higher scores indicating greater frailty.

#### Mediating variables

In this study, sleep quality and anxiety were the mediating variables. Sleep quality was assessed using the Pittsburgh Sleep Quality Index (PSQI) [30]. The PSQI is a validated and reliable instrument for assessing an individual’s sleep quality over the preceding month. It comprises seven dimensions, including daytime dysfunction, sleep latency, habitual sleep efficiency, sleep duration, sleep disorders, use of sleeping pills, and subjective sleep quality. Each dimension is assessed from 0 to 3, resulting in a total score ranging from 0 to 21, with higher scores reflecting poorer sleep quality. The PSQI has acceptable internal consistency, with a Cronbach’s α of 0.714 for the current sample.

Respondents were asked to complete the 7-item Generalized Anxiety Disorder Scale (GAD-7) to evaluate anxiety over the preceding two-week period [31]. Each item on a 4-point Likert scale ranged from “Not at all” (0 points) to “Nearly every day” (3 points). GAD-7 scores range from 0 to 21, with higher scores indicating greater anxiety. The GAD-7 has been demonstrated to have good reliability and acceptable internal consistency by several previous studies [32, 33]. Cronbach’s α of the GAD-7 was 0.945 in the current study.

#### Covariates

Covariates in this study included sociodemographic characteristics (age [continuous variable], sex [male and female], education level [illiterate, primary school, middle school, and high school or above], marital status [have a spouse and no spouse], annual household income [< 50,000 yuan, 50,000–99,999 yuan, 1,00,000–1,49,999 yuan, and ≥ 1,50,000 yuan]), lifestyle habits (smoking status [non-smoker, ex-smoker, and current smoker], current alcohol consumption [no and yes], and dietary habits), and social support. At the first follow-up of the Liyang Study, marital status was divided into four categories: married, unmarried, divorced, and widowed. Married indicated having a spouse, whereas unmarried, divorced, or widowed indicated not having a spouse. Dietary habits were assessed using a food frequency questionnaire (see Supplementary Table 2 for details) [34]. Social support was measured through the question: “Do you have anyone you can share your innermost feelings with and confide in?” (Yes/No).

### Statistical analysis

SAS version 9.4 (SAS Institute Inc., Cary, NC, USA) was utilized for data collection and descriptive analysis, and IBM SPSS version 26.0 (SPSS Inc., Armonk, NY, USA) was used for Spearman correlation and mediation analysis. It was considered statistically significant if the *P* value was less than 0.05.

The analytical process used in the current study was as follows: First, the main characteristics of the study population were described. Categorical variables were presented as numbers (percentages). Given the skewed distribution of age, frailty scores, sleep quality scores, anxiety scores, and multimorbidity scores, the median and interquartile range (IQR) were used to describe these continuous variables. Second, Spearman correlation analysis was employed to examine the correlations between frailty, sleep quality, anxiety, and multimorbidity. Third, a chain mediation analysis of sleep quality and anxiety on the relationship between frailty and multimorbidity was conducted, adjusting age, sex, education level, marital status, annual household income, smoking status, and alcohol consumption status, using the SPSS PROCESS macro (Model 6), as suggested by Hayes et al. [35]. Specifically, after all continuous variables were standardised, the regression coefficients were analysed using bias-corrected bootstrap methods, considering all covariates. The regression coefficient, which was the slope, measured the effect of the independent variable on the dependent variable. Meanwhile, 95% bias-corrected confidence intervals (CIs) were calculated for these effects using 5000 bootstrap samples, with 95% bias-corrected CIs that did not contain zero denoting a significant effect. Finally, to evaluate the robustness of our findings, we adjusted for dietary habits and social support as covariates and conducted sensitivity analyses.

## Results

### Characteristics of participants

The main characteristics of the study participants are presented in Table 1. Among the 2874 participants, ages ranged from 60 to 97 years, with a median age of 70 years (IQR: 65–76). More than half (54.07%) were female, 1124 (39.11%) were illiterate, the overwhelming majority (81.94%) had a spouse, and 1883 (65.52%) had annual household incomes of < 50,000 yuan. The numbers of current smokers and drinkers were 651 (22.65%) and 771 (26.83%), respectively. Frailty, sleep quality, anxiety and multimorbidity scores were 1 (0–1), 5 (3–8), 0 (0–1), and 1 (0–2), respectively.

**Table 1:** Main characteristics of the study participants

Characteristics	*N* (%)	Median (IQR)
Observations	2874 (100.00%)	
Age, years		70 (65, 76)
Sex		
Male	1320 (45.93%)	
Female	1554 (54.07%)	
Education level		
Illiterate	1124 (39.11%)	
Primary school	743 (25.85%)	
Middle school	737 (25.64%)	
High school or above	270 (9.39%)	
Marital status		
Have a spouse	2355 (81.94%)	
No spouse	519 (18.06%)	
Annual household income, yuan		
< 50,000	1883 (65.52%)	
50,000–99,999	560 (19.49%)	
1,00,000–1,49,999	260 (9.05%)	
≥ 1,50,000	171 (5.95%)	
Smoking status		
Non-smoker	2020 (70.29%)	
Ex-smoker	203 (7.06%)	
Current smoker	651 (22.65%)	
Current alcohol consumption		
No	2103 (73.17%)	
Yes	771 (26.83%)	
Dietary habits		
Rice		
Daily	2842 (98.89%)	
4–6 days/week	13 (0.45%)	
1–3 days/week	8 (0.28%)	
Monthly	4 (0.14%)	
Never/rarely	7 (0.24%)	
Wheat		
Daily	184 (6.40%)	
4–6 days/week	235 (8.18%)	
1–3 days/week	1296 (45.09%)	
Monthly	843 (29.33%)	
Never/rarely	316 (11.00%)	
Other staple food (corn, millet, etc.)		
Daily	456 (15.87%)	
4–6 days/week	188 (6.54%)	
1–3 days/week	839 (29.19%)	
Monthly	679 (23.63%)	
Never/rarely	712 (24.77%)	
Meat		
Daily	522 (18.16%)	
4–6 days/week	508 (17.68%)	
1–3 days/week	1352 (47.04%)	
Monthly	345 (12.00%)	
Never/rarely	147 (5.11%)	
Poultry		
Daily	61 (2.12%)	
4–6 days/week	210 (7.31%)	
1–3 days/week	1299 (45.20%)	
Monthly	849 (29.54%)	
Never/rarely	455 (15.83%)	
Fish/seafood		
Daily	98 (3.41%)	
4–6 days/week	298 (10.37%)	
1–3 days/week	1415 (49.23%)	
Monthly	641 (22.30%)	
Never/rarely	422 (14.68%)	
Fresh eggs		
Daily	1428 (49.69%)	
4–6 days/week	523 (18.20%)	
1–3 days/week	658 (22.89%)	
Monthly	140 (4.87%)	
Never/rarely	125 (4.35%)	
Fresh vegetables		
Daily	2788 (97.01%)	
4–6 days/week	50 (1.74%)	
1–3 days/week	25 (0.87%)	
Monthly	3 (0.10%)	
Never/rarely	8 (0.28%)	
Soybean products		
Daily	147 (5.11%)	
4–6 days/week	266 (9.26%)	
1–3 days/week	1143 (39.77%)	
Monthly	752 (26.17%)	
Never/rarely	566 (19.69%)	
Preserved vegetables		
Daily	509 (17.71%)	
4–6 days/week	240 (8.35%)	
1–3 days/week	627 (21.82%)	
Monthly	641 (22.30%)	
Never/rarely	857 (29.82%)	
Fresh fruit		
Daily	801 (27.87%)	
4–6 days/week	385 (13.40%)	
1–3 days/week	762 (26.51%)	
Monthly	525 (18.27%)	
Never/rarely	401 (13.95%)	
Dairy products (milk, yoghurt)		
Daily	510 (17.75%)	
4–6 days/week	210 (7.31%)	
1–3 days/week	438 (15.24%)	
Monthly	484 (16.84%)	
Never/rarely	1232 (42.87%)	
Social support		
No	319 (11.10%)	
Yes	2555 (88.90%)	
Frailty, score		1 (0, 1)
Sleep quality, score		5 (3, 8)
Anxiety, score		0 (0, 1)
Multimorbidity, score		1 (0, 2)

*N (%)* Frequency (percentage), *IQR* Interquartile range

### Correlation analysis

Supplementary Table 3 shows the significant correlations between all four core variables. Specifically, frailty was positively correlated with sleep quality, anxiety, and multimorbidity (*P* < 0.01). Sleep quality was positively related to anxiety and multimorbidity (*P* < 0.01). And, anxiety and multimorbidity were positively associated (*P* < 0.01).

### Mediating effects of sleep quality and anxiety

After adjustment for all covariates, frailty positively predicted sleep quality, anxiety, and multimorbidity (*β* = 0.806, 0.877, and 0.093, respectively; *P* < 0.001 for all). Sleep quality positively predicted anxiety and multimorbidity (*β* = 0.258 and 0.065, respectively; all *P* < 0.001). Anxiety positively predicted multimorbidity (*β* = 0.047, *P* < 0.001) (Table 2).

**Table 2:** Results of regression analysis of the chain mediation effects model

Variables	Sleep quality				Anxiety				Multimorbidity			
	*β*	SE	t	*P*	*β*	SE	t	*P*	*β*	SE	t	*P*
Constant	2.457**	0.879	2.797	< 0.01	−1.194	0.673	−1.773	0.076	0.783**	0.301	2.603	< 0.01
Age	0.009	0.010	0.868	0.386	−0.012	0.008	−1.560	0.119	0.001	0.003	0.295	0.768
Sex	0.760***	0.188	4.052	< 0.001	0.393**	0.144	2.726	< 0.01	−0.233***	0.064	−3.609	< 0.001
Annual household income	−0.085	0.076	−1.125	0.261	−0.042	0.058	−0.717	0.474	−0.005	0.026	−0.201	0.841
Smoking status	0.235*	0.105	2.251	< 0.05	−0.015	0.080	−0.181	0.857	0.094**	0.036	2.619	< 0.01
Drinking status	0.199	0.170	1.169	0.243	0.231	0.130	1.771	0.077	0.146*	0.058	2.507	< 0.05
Education level	0.042	0.073	0.574	0.566	0.013	0.056	0.228	0.819	−0.003	0.025	−0.136	0.892
Marital status	−0.372*	0.182	−2.049	< 0.05	0.166	0.139	1.191	0.234	−0.081	0.062	−1.303	0.193
Frailty	0.806***	0.072	11.202	< 0.001	0.877***	0.056	15.605	< 0.001	0.093***	0.026	3.541	< 0.001
Sleep quality					0.258***	0.014	18.014	< 0.001	0.065***	0.007	9.640	< 0.001
Anxiety									0.047***	0.008	5.631	< 0.001
R	0.284				0.469				0.304			
R-square	0.081				0.220				0.092			
*P*	< 0.001				< 0.001				< 0.001			

The model was adjusted for age, sex, education level, marital status, annual household income, smoking status, and alcohol consumption

*SE* Standard error

***P < 0.001; **P < 0.01; *P < 0.05

Table 3 presents the mediating effects of sleep quality and anxiety on the association between frailty and multimorbidity, after adjusting for all covariates. The results indicated a significant total effect of frailty on multimorbidity (unstandardised coefficient [B] = 0.196, bootstrap 95% CI = [0.146, 0.246]), with the direct effect of frailty on multimorbidity accounting for 47.45% of the total effect (B = 0.093, bootstrap 95% CI = [0.041, 0.144]). The total mediating effect, which was stronger than the direct effect, comprised 52.55% of the total effect (B = 0.103, bootstrap 95% CI = [0.079, 0.130]). Of the mediation effects, sleep quality and anxiety both significantly mediated the association between frailty and multimorbidity (B = 0.052, bootstrap 95% CI = [0.038, 0.068]; B = 0.041, bootstrap 95% CI = [0.025, 0.060]), contributing 26.53% and 20.92% of the mediating effect, respectively. The chained mediating effects of sleep quality and anxiety on the relationship between frailty and multimorbidity were also significant, representing 5.10% of the total mediating effect (B = 0.010, bootstrap 95% CI = [0.006, 0.015]).

**Table 3:** Bootstrap analysis of total, direct, and indirect effects

Model pathways	B	SE	Boot LLCI	Boot ULCI	Effectiveness ratio %
Total effect					
X → Y	0.196	0.025	0.146	0.246	100.00
Direct effect					
X → Y	0.093	0.026	0.041	0.144	47.45
Total indirect effect	0.103	0.013	0.079	0.130	52.55
X → M1 → Y	0.052	0.008	0.038	0.068	26.53
X → M2 → Y	0.041	0.009	0.025	0.060	20.92
X → M1 → M2 → Y	0.010	0.002	0.006	0.015	5.10

The model was adjusted for age, sex, education level, marital status, annual household income, smoking status, and alcohol consumption

*B* Unstandardised coefficient, *SE* Standard error, *Boot LLCI* Bootstrap lower limit confidence interval, *Boot ULCI* Bootstrap upper limit confidence interval, *X* Frailty, *Y* Multimorbidity, *M1* Sleep quality, *M2* Anxiety

**Fig. 3:**
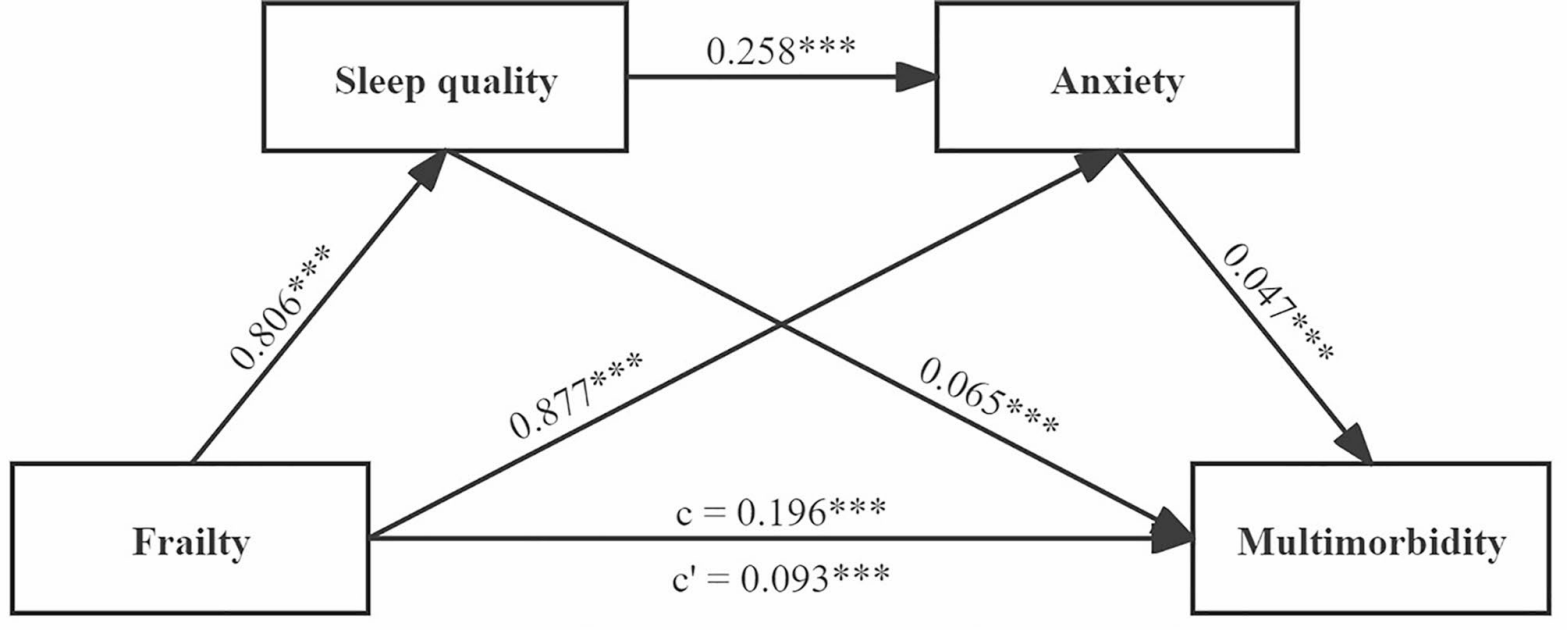
A chain mediation model of sleep quality and anxiety on the relationship between frailty and multimorbidity. Notes: ^***^P < 0.001; ^**^P < 0.01; ^*^P < 0.05. Path c represents the total effect and path c’ indicates the direct effect. The model adjusted for age, sex, education level, marital status, annual household income, smoking status, and alcohol consumption

Finally, the specific pathways of the chain mediation model of sleep quality and anxiety from frailty to multimorbidity are summarised in Fig. 3.

### Sensitivity analyses

Sensitivity analyses demonstrated robustness, with the chain mediation model adjusting for dietary habits and social support producing results consistent with our main findings (*P* < 0.001) (Supplementary Table 4).

## Discussion

Based on the first follow-up of the Liyang Study, this study confirmed the direct positive association between frailty and multimorbidity in a sample of Chinese individuals aged ≥60 years, while also revealing that sleep quality and anxiety partially mediate this relationship. Furthermore, research has demonstrated a significant association between sleep quality and anxiety, which subsequently affects multimorbidity. This study offers novel insights into how frailty affects multimorbidity in elderly individuals.

This study confirmed that frailty has a direct effect on multimorbidity in the elderly population, with an effect size of 47.45%. This discovery provides further support for Hypothesis 1, which was corroborated in previous studies [10, 36]. A study of Chinese older adults found that frailty was positively associated with multimorbidity [36]. This has several possible interpretations. On the one hand, inflammatory biomarkers may serve as the bridge between the association. A study conducted in community-dwelling men older than 75 years in Australia indicates a potential cross-sectional association between interleukin-6 and interleukin-8 and frailty [37]. In a 9-year longitudinal study, Fabbri et al. concluded that higher baseline interleukin-6 and a sharp increase in interleukin-6 levels are independently and significantly associated with multimorbidity in Italian participants aged 60 years or over [38]. On the other hand, one potential explanation is that the physiological systems of older individuals with frailty render them more vulnerable to various stressors, heightening the likelihood of adverse health consequences resulting from an impaired capacity to maintain homeostasis, ultimately culminating in multimorbidity [39, 40]. Therefore, the identification of populations at heightened risk of multimorbidity is feasible by promptly recognising frailty. The implementation of suitable interventions for special populations enhances the efficient allocation of resources to prevent multimorbidity.

The total mediated effect accounted for 52.55% of the relationship between frailty and multimorbidity in the present study, which surpassed the direct effect. These results indicate the significance of mediators, including sleep quality and anxiety, in the association between frailty and multimorbidity. The mediation model proposed in this study suggests that sleep quality mediates the association between frailty and multimorbidity, with a mediation effect of 26.53%. Consequently, Hypothesis 2 was confirmed. This assertion is supported by the results of previous studies [41–45]. Frailty may result in disturbed sleep rhythms, decreased activity, and fatigue that contribute to increased drowsiness and further compromise sleep quality [41, 42]. A study conducted in Japan by Maekawa et al. suggests that pre-frail individuals may fall asleep later than non-frail individuals [46]. Leptin, an important adipokine, may play a crucial role in this process. Leptin levels are low in older adults with frailty [47]. It maintains deep sleep by antagonizing hypothalamic orexin neurons [48]. Poor sleep quality has the potential to affect multimorbidity by interfering with several physiological pathways, including the disruption of cardiometabolic, endocrine, immune, and inflammatory functions [43]. Specifically, irregular sleep patterns lead to physiological changes, such as autonomic nervous dysfunction, which can cause increased blood pressure, insulin resistance, and abnormal lipid profile [49]. Sleep deprivation was associated with elevated levels of nocturnal catecholamine, which may cause cardiovascular disease [50]. In addition, previous studies have established that sleep disorders can lead to the dysregulation of inflammatory and antiviral responses characterized by increased C-reactive protein, ultimately leading to compromised immune defences, which may explain their effects on multimorbidity [44]. Notably, our findings indicated that sleep quality was the most influential mediating factor in the relationship between frailty and multimorbidity, underscoring the need to enhance sleep quality among elderly individuals with frailty.

Moreover, the results of this study suggest that anxiety plays a mediating role in the association between frailty and multimorbidity in older adults, with a mediating effect of 20.92%. Thus, older adults with frailty were more likely to experience multimorbidity owing to anxiety, validating Hypothesis 3. In the former half of the mediating relationship, a positive association was observed between frailty and anxiety, aligning with findings from prior studies [51]. A population-based cross-sectional study conducted in Dublin, Ireland, involving individuals aged ≥ 60 years observed that people with pre-frailty and frailty had higher anxiety scores than robust older adults [52]. This phenomenon could potentially be attributed to various driving factors of frailty, such as smoking, insufficient physical activity, obesity, or malnutrition, which can result in diminished functionality, increased susceptibility to disability, and consequently anxiety [19]. Hence, the incorporation of frailty monitoring and assessment into existing routine medical examinations for elderly adults can aid in the detection of anxiety. In the latter half of the mediating relationship, anxiety was positively associated with multimorbidity, which is similar to previous studies [53]. Farooq et al. proposed that mental illness is associated with unhealthy lifestyle behaviours, including smoking, inadequate self-care, poor medication adherence, and lack of physical activity, potentially elucidating the impact of anxiety on multimorbidity [54]. In addition, the overexpression of certain biological pathways in individuals with anxiety has been shown to be associated with multimorbidity, initiated by the cumulative effects of stress [17]. Therefore, the prioritisation of mental health in older individuals with frailty may be an effective method for public health professionals to prevent multimorbidity.

The present study also revealed that the effect of frailty on multimorbidity in older adults was chain-mediated through poor sleep quality and anxiety, with a mediating effect of 5.10%, thereby confirming Hypothesis 4. In other words, frailty is positively associated with poor sleep quality, resulting in increased anxiety and ultimately contributing to multimorbidity. In addition, a powerful systematic review pointed out that sleep disturbance is a risk factor for anxiety [55]. Physiologically, the anxiolytic impact of sleep deprivation is associated with impaired activity in the medial prefrontal cortex and connections to extended limbic regions [56]. Therefore, improving sleep quality and mitigating psychological issues in older adults experiencing frailty may be suitable approaches for preventing multimorbidity.

Our study had several limitations. First, all variables were obtained through self-reporting, except for some items that constituted frailty, which relied on objective measures, potentially introducing recall bias. However, participants were supported by a trained physician when providing these data and self-reported diseases have been acknowledged as a reliable indicator of prevalence [57]. The PSQI and GAD-7 have acceptable internal consistency in our study. Second, the recruitment of study participants exclusively from Liyang City, China, may restrict the generalisability of the findings to a broader population. Further studies in multiple countries or regions are required to validate our results. Third, a causal association between frailty, sleep quality, anxiety, and multimorbidity could not be derived due to the cross-sectional design. Longitudinal and prospective studies are essential to adequately address this issue. Fourth, other potential mediators should be considered in future studies. Fifth, despite extensive adjustment for potential confounding factors, the possibility of residual confounding remains. Finally, our examination was limited to the mediating pathways outlined earlier, neglecting alternative pathways. Consequently, additional potential mechanisms warrant further exploration in subsequent research endeavours.

## Conclusions

In conclusion, frailty was positively associated with multimorbidity in older Chinese individuals, and sleep quality and anxiety were important mediators of the association between frailty and multimorbidity. Therefore, preventing or reversing frailty, adopting high-quality sleep patterns, and focusing on the mental well-being of older adults can serve as effective strategies for mitigating the development of multimorbidity, thereby enhancing the overall health and quality of life of this population. For example, music therapy can be a cost-effective intervention to improve sleep quality [58]. The findings of this study offer guidance for future investigations aimed at elucidating the association between frailty and multimorbidity in elderly populations, potentially contributing to the development of more effective strategies to prevent multimorbidity.

## Supplementary Information

Supplementary Material 1.

## Data Availability

The datasets used and analysed during the current study are available from the corresponding author on reasonable request.

## Abbreviations

B: Unstandardised coefficient
CHARLS: China Health and Retirement Longitudinal Study
CI: Confidence interval
GAD-7: 7-item Generalized Anxiety Disorder Scale
IQR: Interquartile range
PSQI: Pittsburgh Sleep Quality Index

## References

[1] Valderas JM, Starfield B, Sibbald B, Salisbury C, Roland M. Defining comorbidity: implications for Understanding health and health services. Ann Fam Med. 2009;7(4):357–63. 10.1370/afm.983. 19597174 10.1370/afm.983 PMC2713155

[2] Yao SS, Cao GY, Han L, Chen ZS, Huang ZT, Gong P, Hu Y, Xu B. Prevalence and patterns of Multimorbidity in a nationally representative sample of older chinese: results from the China health and retirement longitudinal study. J Gerontol Biol Sci Med Sci. 2020;75(10):1974–80. 10.1093/gerona/glz185. 10.1093/gerona/glz185 31406983

[3] Vogeli C, Shields AE, Lee TA, Gibson TB, Marder WD, Weiss KB, Blumenthal D. Multiple chronic conditions: prevalence, health consequences, and implications for quality, care management, and costs. J Gen Intern Med. 2007;22(Suppl 3):391–5. 10.1007/s11606-007-0322-1. 18026807 10.1007/s11606-007-0322-1 PMC2150598

[4] Menotti A, Mulder I, Nissinen A, Giampaoli S, Feskens EJ, Kromhout D. Prevalence of morbidity and Multimorbidity in elderly male populations and their impact on 10-year all-cause mortality: the FINE study (Finland, Italy, Netherlands, Elderly). J Clin Epidemiol. 2001;54(7):680–6. 10.1016/s0895-4356(00)00368-1. 11438408 10.1016/s0895-4356(00)00368-1

[5] Fortin M, Lapointe L, Hudon C, Vanasse A, Ntetu AL, Maltais D. Multimorbidity and quality of life in primary care: a systematic review. Health Qual Life Outcomes. 2004;2:51. 10.1186/1477-7525-2-51. 15380021 10.1186/1477-7525-2-51 PMC526383

[6] Huang ST, Tange C, Otsuka R, Nishita Y, Peng LN, Hsiao FY, Tomida M, Shimokata H, Arai H, Chen LK. Subtypes of physical frailty and their long-term outcomes: a longitudinal cohort study. J Cachexia Sarcopenia Muscle. 2020;11(5):1223–31. 10.1002/jcsm.12577. 32558267 10.1002/jcsm.12577 PMC7567152

[7] Guaraldi G, Brothers TD, Zona S, Stentarelli C, Carli F, Malagoli A, Santoro A, Menozzi M, Mussi C, Mussini C, et al. A frailty index predicts survival and incident Multimorbidity independent of markers of HIV disease severity. AIDS. 2015;29(13):1633–41. 10.1097/QAD.0000000000000753. 26372273 10.1097/QAD.0000000000000753

[8] Rodriguez-Manas L, Feart C, Mann G, Vina J, Chatterji S, Chodzko-Zajko W, Gonzalez-Colaco Harmand M, Bergman H, Carcaillon L, Nicholson C, et al. Searching for an operational definition of frailty: a Delphi method based consensus statement: the frailty operative definition-consensus conference project. J Gerontol Biol Sci Med Sci. 2013;68(1):62–7. 10.1093/gerona/gls119. 10.1093/gerona/gls119 PMC3598366 22511289

[9] Hoogendijk EO, Afilalo J, Ensrud KE, Kowal P, Onder G, Fried LP. Frailty: implications for clinical practice and public health. Lancet. 2019;394(10206):1365–75. 10.1016/S0140-6736(19)31786-6. 31609228 10.1016/S0140-6736(19)31786-6

[10] Hanlon P, Nicholl BI, Jani BD, Lee D, McQueenie R, Mair FS. Frailty and pre-frailty in middle-aged and older adults and its association with Multimorbidity and mortality: a prospective analysis of 493 737 UK biobank participants. Lancet Public Health. 2018;3(7):e323–32. 10.1016/S2468-2667(18)30091-4. 29908859 10.1016/S2468-2667(18)30091-4 PMC6028743

[11] Ferrucci L, Fabbri E. Inflammageing: chronic inflammation in ageing, cardiovascular disease, and frailty. Nat Rev Cardiol. 2018;15(9):505–22. 10.1038/s41569-018-0064-2. 30065258 10.1038/s41569-018-0064-2 PMC6146930

[12] Zhong HH, Yu B, Luo D, Yang LY, Zhang J, Jiang SS, Hu SJ, Luo YY, Yang MW, Hong FF, Yang SL. Roles of aging in sleep. Neurosci Biobehav Rev. 2019;98:177–84. 10.1016/j.neubiorev.2019.01.013. 30648559 10.1016/j.neubiorev.2019.01.013

[13] Nistor P, Chang-Kit B, Nicholson K, Anderson KK, Stranges S. The relationship between sleep health and Multimorbidity in community dwelling populations: systematic review and global perspectives. Sleep Med. 2023;109:270–84. 10.1016/j.sleep.2023.07.002. 37490803 10.1016/j.sleep.2023.07.002

[14] Zhou Y, Ni Y, Jones M, Dai X, Lim CCW, Zhu A, Xu X. Sleep behaviors and progression of Multimorbidity in Middle-Aged and older adults: A prospective cohort study from China. J Gerontol Biol Sci Med Sci. 2023;78(10):1871–80. 10.1093/gerona/glad087. 10.1093/gerona/glad087 36943283

[15] Zou C, Sun H, Lu C, Chen W, Guo VY. Nighttime sleep duration, restlessness and risk of multimorbidity - A longitudinal study among middle-aged and older adults in China. Arch Gerontol Geriatr. 2022;99:104580. 10.1016/j.archger.2021.104580. 34837791 10.1016/j.archger.2021.104580

[16] Xu X, Zhou X, Liu W, Ma Q, Deng X, Fang R. Evaluation of the correlation between frailty and sleep quality among elderly patients with osteoporosis: a cross-sectional study. BMC Geriatr. 2022;22(1):599. 10.1186/s12877-022-03285-z. 35854210 10.1186/s12877-022-03285-z PMC9295528

[17] Bobo WV, Grossardt BR, Virani S, St Sauver JL, Boyd CM, Rocca WA. Association of depression and anxiety with the accumulation of chronic conditions. JAMA Netw Open. 2022;5(5):e229817. 10.1001/jamanetworkopen.2022.9817. 35499825 10.1001/jamanetworkopen.2022.9817 PMC9062691

[18] Braude P, McCarthy K, Strawbridge R, Short R, Verduri A, Vilches-Moraga A, Hewitt J, Carter B. Frailty is associated with poor mental health 1 year after hospitalisation with COVID-19. J Affect Disord. 2022;310:377–83. 10.1016/j.jad.2022.05.035. 35568322 10.1016/j.jad.2022.05.035 PMC9091159

[19] Tan M, Bhanu C, Frost R. The association between frailty and anxiety: A systematic review. Int J Geriatr Psychiatry. 2023;38(5):e5918. 10.1002/gps.5918. 37157226 10.1002/gps.5918

[20] Peng A, Ji S, Lai W, Hu D, Wang M, Zhao X, Chen L. The bidirectional relationship between sleep disturbance and anxiety: sleep disturbance is a stronger predictor of anxiety. Sleep Med. 2024;121:63–8. 10.1016/j.sleep.2024.06.022. 38924831 10.1016/j.sleep.2024.06.022

[21] Zhou L, Hu W, Liu S, Qiao Y, He D, Xiong S, Peng L, Cao L, Wu Y, Sun N, et al. Cohort profile: the Liyang cohort study on chronic diseases and risk factors monitoring in China (Liyang Study). BMJ Open. 2022;12(7):e060978. 10.1136/bmjopen-2022-060978. 35851009 10.1136/bmjopen-2022-060978 PMC9297217

[22] Shi Z, Zhang Z, Shi K, Yu B, Jiang Z, Yang L, Lin J, Fang Y. Association between Multimorbidity trajectories and incident disability among mid to older age adults: China health and retirement longitudinal study. BMC Geriatr. 2022;22(1):741. 10.1186/s12877-022-03421-9. 36096760 10.1186/s12877-022-03421-9 PMC9469590

[23] Ben Hassen C, Fayosse A, Landre B, Raggi M, Bloomberg M, Sabia S, Singh-Manoux A. Association between age at onset of Multimorbidity and incidence of dementia: 30 year follow-up in Whitehall II prospective cohort study. BMJ. 2022;376:e068005. 10.1136/bmj-2021-068005. 35110302 10.1136/bmj-2021-068005 PMC9086721

[24] Jiang CH, Zhu F, Qin TT. Relationships between chronic diseases and depression among Middle-aged and elderly people in china: A prospective study from CHARLS. Curr Med Sci. 2020;40(5):858–70. 10.1007/s11596-020-2270-5. 33123901 10.1007/s11596-020-2270-5

[25] Vetrano DL, Calderon-Larranaga A, Marengoni A, Onder G, Bauer JM, Cesari M, Ferrucci L, Fratiglioni L. An international perspective on chronic multimorbidity: approaching the elephant in the room. J Gerontol Biol Sci Med Sci. 2018;73(10):1350–6. 10.1093/gerona/glx178. 10.1093/gerona/glx178 PMC6132114 28957993

[26] Fried LP, Tangen CM, Walston J, Newman AB, Hirsch C, Gottdiener J, Seeman T, Tracy R, Kop WJ, Burke G, et al. Frailty in older adults: evidence for a phenotype. J Gerontol Biol Sci Med Sci. 2001;56(3):M146–156. 10.1093/gerona/56.3.m146. 10.1093/gerona/56.3.m146 11253156

[27] Wu C, Smit E, Xue QL, Odden MC. Prevalence and correlates of frailty among Community-Dwelling Chinese older adults: the China health and retirement longitudinal study. J Gerontol Biol Sci Med Sci. 2017;73(1):102–8. 10.1093/gerona/glx098. 10.1093/gerona/glx098 PMC5861883 28525586

[28] Xu W, Li YX, Wu C. Incidence of frailty among community-dwelling older adults: a nationally representative profile in China. BMC Geriatr. 2019;19(1):378. 10.1186/s12877-019-1393-7. 31888498 10.1186/s12877-019-1393-7 PMC6937935

[29] Yuan M, Xu C, Fang Y. The transitions and predictors of cognitive frailty with multi-state Markov model: a cohort study. BMC Geriatr. 2022;22(1):550. 10.1186/s12877-022-03220-2. 35778705 10.1186/s12877-022-03220-2 PMC9248089

[30] Buysse DJ, Reynolds CF 3rd, Monk TH, Berman SR, Kupfer DJ. The Pittsburgh sleep quality index: a new instrument for psychiatric practice and research. Psychiatry Res. 1989;28(2):193–213. 10.1016/0165-1781(89)90047-4. 2748771 10.1016/0165-1781(89)90047-4

[31] Spitzer RL, Kroenke K, Williams JB, Lowe B. A brief measure for assessing generalized anxiety disorder: the GAD-7. Arch Intern Med. 2006;166(10):1092–7. 10.1001/archinte.166.10.1092. 16717171 10.1001/archinte.166.10.1092

[32] Ji K, Bai Z, Tang L, Yan H, Zhu Y, Chen G, Chen R. Institutional satisfaction and anxiety mediate the relationship between social support and depression in hypertension patients in elderly caring social organizations: A Cross-Sectional study. Front Psychol. 2021;12:772092. 10.3389/fpsyg.2021.772092. 34759876 10.3389/fpsyg.2021.772092 PMC8573192

[33] Zhao L, Xu F, Zheng X, Xu Z, Osten B, Ji K, Ding S, Liu G, Yang S, Chen R. Mediation role of anxiety on social support and depression among diabetic patients in elderly caring social organizations in China during COVID-19 pandemic: a cross-sectional study. BMC Geriatr. 2023;23(1):790. 10.1186/s12877-023-04502-z. 38041007 10.1186/s12877-023-04502-z PMC10691130

[34] Qin C, Guo Y, Pei P, Du H, Yang L, Chen Y, Shen X, Shi Z, Qi L, Chen J, et al. The relative validity and reproducibility of food frequency questionnaires in the China kadoorie biobank study. Nutrients. 2022;14(4). 10.3390/nu14040794. 10.3390/nu14040794 PMC8879142 35215443

[35] Hayes AF. Introduction to Mediation, Moderation, and conditional process analysis: A Regression-Based approach. New York: Guilford Press; 2013.

[36] Yuan Y, Li J, Fu P, Zhou C, Li S. Association between frailty and inpatient services utilization among older adults in rural china: the mediating role of Multimorbidity. Front Med (Lausanne). 2022;9:818482. 10.3389/fmed.2022.818482. 35178412 10.3389/fmed.2022.818482 PMC8844457

[37] Hsu B, Hirani V, Cumming RG, Naganathan V, Blyth FM, Wright FC, Waite LM, Seibel MJ, Handelsman DJ, Le Couteur DG. Cross-Sectional and longitudinal relationships between inflammatory biomarkers and frailty in Community-dwelling older men: the concord health and ageing in men project. J Gerontol Biol Sci Med Sci. 2019;74(6):835–41. 10.1093/gerona/glx142. 10.1093/gerona/glx142 28977375

[38] Fabbri E, An Y, Zoli M, Simonsick EM, Guralnik JM, Bandinelli S, Boyd CM, Ferrucci L. Aging and the burden of multimorbidity: associations with inflammatory and anabolic hormonal biomarkers. J Gerontol Biol Sci Med Sci. 2015;70(1):63–70. 10.1093/gerona/glu127. 10.1093/gerona/glu127 PMC4296167 25104822

[39] Clegg A, Young J, Iliffe S, Rikkert MO, Rockwood K. Frailty in elderly people. Lancet. 2013;381(9868):752–62. 10.1016/S0140-6736(12)62167-9. 23395245 10.1016/S0140-6736(12)62167-9 PMC4098658

[40] Villacampa-Fernandez P, Navarro-Pardo E, Tarin JJ, Cano A. Frailty and multimorbidity: two related yet different concepts. Maturitas. 2017;95:31–5. 10.1016/j.maturitas.2016.10.008. 27889050 10.1016/j.maturitas.2016.10.008

[41] Cavusoglu C, Deniz O, Tuna Dogrul R, Coteli S, Oncul A, Kizilarslanoglu MC, Gcker B. Frailty is associated with poor sleep quality in the oldest old. Turk J Med Sci. 2021;51(2):540–6. 10.3906/sag-2001-168. 32950043 10.3906/sag-2001-168 PMC8203150

[42] Mander BA, Winer JR, Walker MP, Sleep, Aging H. Neuron. 2017;94(1):19–36. 10.1016/j.neuron.2017.02.004. 28384471 10.1016/j.neuron.2017.02.004 PMC5810920

[43] Nicholson K, Rodrigues R, Anderson KK, Wilk P, Guaiana G, Stranges S. Sleep behaviours and Multimorbidity occurrence in middle-aged and older adults: findings from the Canadian longitudinal study on aging (CLSA). Sleep Med. 2020;75:156–62. 10.1016/j.sleep.2020.07.002. 32858355 10.1016/j.sleep.2020.07.002

[44] Irwin MR. Sleep and inflammation: partners in sickness and in health. Nat Rev Immunol. 2019;19(11):702–15. 10.1038/s41577-019-0190-z. 31289370 10.1038/s41577-019-0190-z

[45] Kyprianidou M, Panagiotakos D, Kambanaros M, Makris KC, Christophi CA. Quality of sleep in the Cypriot population and its association with multimorbidity: A Cross-Sectional study. Front Public Health. 2021;9:693332. 10.3389/fpubh.2021.693332. 34778165 10.3389/fpubh.2021.693332 PMC8585989

[46] Maekawa H, Kume Y. Imbalance of nonparametric rest-activity rhythm and the evening-type of chronotype according to frailty indicators in elderly community dwellers. Chronobiol Int. 2019;36(9):1208–16. 10.1080/07420528.2019.1626416. 31234663 10.1080/07420528.2019.1626416

[47] Hubbard RE, O’Mahony MS, Calver BL, Woodhouse KW. Nutrition, inflammation, and leptin levels in aging and frailty. J Am Geriatr Soc. 2008;56(2):279–84. 10.1111/j.1532-5415.2007.01548.x. 18179487 10.1111/j.1532-5415.2007.01548.x

[48] Hirota T, Morioka T, Yoda K, Toi N, Hayashi N, Maruo S, Yamazaki Y, Kurajoh M, Motoyama K, Yamada S, et al. Positive association of plasma leptin with sleep quality in obese type 2 diabetes patients. J Diabetes Investig. 2018;9(5):1100–5. 10.1111/jdi.12826. 29479862 10.1111/jdi.12826 PMC6123027

[49] Huang T, Redline S. Cross-sectional and prospective associations of Actigraphy-Assessed sleep regularity with metabolic abnormalities: the Multi-Ethnic study of atherosclerosis. Diabetes Care. 2019;42(8):1422–9. 10.2337/dc19-0596. 31167888 10.2337/dc19-0596 PMC6647049

[50] Irwin M, Thompson J, Miller C, Gillin JC, Ziegler M. Effects of sleep and sleep deprivation on catecholamine and interleukin-2 levels in humans: clinical implications. J Clin Endocrinol Metab. 1999;84(6):1979–85. 10.1210/jcem.84.6.5788. 10372697 10.1210/jcem.84.6.5788

[51] Bernal-Lopez C, Potvin O, Avila-Funes JA. Frailty is associated with anxiety in community-dwelling elderly adults. J Am Geriatr Soc. 2012;60(12):2373–4. 10.1111/jgs.12014. 23231561 10.1111/jgs.12014

[52] Ni Mhaolain AM, Fan CW, Romero-Ortuno R, Cogan L, Cunningham C, Kenny RA, Lawlor B. Frailty, depression, and anxiety in later life. Int Psychogeriatr. 2012;24(8):1265–74. 10.1017/S1041610211002110. 22333477 10.1017/S1041610211002110

[53] Winkler P, Horacek J, Weissova A, Sustr M, Brunovsky M. Physical comorbidities in depression Co-Occurring with anxiety: A cross sectional study in the Czech primary care system. Int J Environ Res Public Health. 2015;12(12):15728–38. 10.3390/ijerph121215015. 26690458 10.3390/ijerph121215015 PMC4690951

[54] Farooq S, Khan T, Zaheer S, Shafique K. Prevalence of anxiety and depressive symptoms and their association with Multimorbidity and demographic factors: a community-based, cross-sectional survey in Karachi, Pakistan. BMJ Open. 2019;9(11):e029315. 10.1136/bmjopen-2019-029315. 31748286 10.1136/bmjopen-2019-029315 PMC6887067

[55] Palmer CA, Bower JL, Cho KW, Clementi MA, Lau S, Oosterhoff B, Alfano CA. Sleep loss and emotion: A systematic review and meta-analysis of over 50 years of experimental research. Psychol Bull. 2024;150(4):440–63. 10.1037/bul0000410. 38127505 10.1037/bul0000410

[56] Ben Simon E, Rossi A, Harvey AG, Walker MP. Overanxious and underslept. Nat Hum Behav. 2020;4(1):100–10. 10.1038/s41562-019-0754-8. 31685950 10.1038/s41562-019-0754-8

[57] Horton M, Rudick RA, Hara-Cleaver C, Marrie RA. Validation of a self-report comorbidity questionnaire for multiple sclerosis. Neuroepidemiology. 2010;35(2):83–90. 10.1159/000311013. 20551692 10.1159/000311013

[58] Loewy J. Music therapy as a potential intervention for sleep improvement. Nat Sci Sleep. 2020;12:1–9. 10.2147/NSS.S194938. 32021519 10.2147/NSS.S194938 PMC6954684

