# Supplementary material for: The relationship between frailty and multimorbidity in Chinese older adults: the chain mediating effects of sleep quality and anxiety
Source: BMC Geriatr. 2025 Oct 27;25:814. doi: 10.1186/s12877-025-06490-8 (PMC12557892; doi:10.1186/s12877-025-06490-8)
Supplement: Supplementary file 1 — Supplementary Material 1. [file 12877_2025_6490_MOESM1_ESM.docx]

**Supplementary Material**

**Supplementary Table 1** The list of frailty definitions.

**Supplementary Table 2** Food frequency questionnaire used in the first follow-up of the Liyang cohort study on chronic diseases and risk factors monitoring in China.

**Supplementary Table 3** Correlations for frailty, sleep quality, anxiety, and multimorbidity.

**Supplementary Table 4** Bootstrap analysis of total, direct, and indirect effects with additional adjustments for dietary habits and social support.

**Supplementary Table 1** The list of frailty definitions.

| Components | Definition |
| --- | --- |
| Weakness | Weakness was defined, using the maximum handgrip strength of either hand (two trials for each; measured in a standing position), as ≤ 20^th^ percentile of the weighted population distribution, adjusting for sex and body mass index (BMI).  Women:  ≤15.0 kg for BMI ≤ 20.0 kg/$m^{2}$  ≤17.5 kg for BMI 20.0 – 22.1 kg/$m^{2}$  ≤17.5 kg for BMI 22.1 – 24.8 kg/$m^{2}$  ≤20.0 kg for BMI > 24.8 kg/$m^{2}$  Men:  ≤25.2 kg for BMI ≤ 20.6 kg/$m^{2}$  ≤28.5 kg for BMI 20.6 – 23.2 kg/$m^{2}$  ≤30.0 kg for BMI 23.2 – 25.9 kg/$m^{2}$  ≤30.0 kg for BMI > 25.9 kg/$m^{2}$ |
| Slowness | Slowness was defined, using the average of two-timed walk tests over a 2.5-meter course, as being ≤20th percentile of the weighted population distribution, adjusting for sex and height.  Women:  ≤0.36 m/s for height ≤151 cm  ≤0.43 m/s for height >151 cm  Men:  ≤0.45 m/s for height ≤163 cm  ≤0.48 m/s for height >163 cm |
| Exhaustion | Exhaustion was characterized by two questions from the modified 10-item Center for Epidemiological Studies-Depression scale [1]. Two questions were, “I could not get going” and “I felt everything I did was an effort”. Participants were asked to indicate the frequency they felt that way during the last week: “rarely/none of the time; less than 1 day” (coded 0), “some or a little of the time; 1 to 2 days” (coded 1), “a moderate amount of time; 3 to 4 days” (coded 2), or “most of the time” (coded 3). Participants met the criteria for exhaustion if they had a total score ≥4. |
| Inactivity | Participants who self-report that they did not walk ≥10 minutes continuously during a usual week are considered inactivity. |
| Shrinking | Shrinking was identified as self-reporting loss of ≥5 kg in the previous year or having a body mass index ≤18.5 kg/$m^{2}$. |

**Supplementary Table 2** Food frequency questionnaire used in the first follow-up of the Liyang cohort study on chronic diseases and risk factors monitoring in China.

**During the past 12 months, how often did you eat the following foods?**

|  | Daily | 4-6 days/week | 1-3 days/week | Monthly | Never/rarely |
| --- | --- | --- | --- | --- | --- |
| Rice | □ | □ | □ | □ | □ |
| Wheat | □ | □ | □ | □ | □ |
| Other staple foods (corn, millet, etc.) | □ | □ | □ | □ | □ |
| Meat | □ | □ | □ | □ | □ |
| Poultry | □ | □ | □ | □ | □ |
| Fish/seafood | □ | □ | □ | □ | □ |
| Fresh eggs | □ | □ | □ | □ | □ |
| Fresh vegetables | □ | □ | □ | □ | □ |
| Soybean products | □ | □ | □ | □ | □ |
| Preserved vegetables | □ | □ | □ | □ | □ |
| Fresh fruit | □ | □ | □ | □ | □ |
| Dairy products (milk, yoghurt) | □ | □ | □ | □ | □ |

**Supplementary Table 3** Correlations for frailty, sleep quality, anxiety, and multimorbidity.

| **Variables** | **1** | **2** | **3** | **4** |
| --- | --- | --- | --- | --- |
| 1. Frailty | 1.000 |  |  |  |
| 2. Sleep quality | 0.151** | 1.000 |  |  |
| 3. Anxiety | 0.203** | 0.370** | 1.000 |  |
| 4. Multimorbidity | 0.142** | 0.237** | 0.206** | 1.000 |

Notes: ****P*<0.001; ***P*<0.01; **P*<0.05.

**Supplementary Table 4** Bootstrap analysis of total, direct, and indirect effects with additional adjustments for dietary habits and social support.

| **Model pathways** | **B** | **SE** | **Boot LLCI** | **Boot ULCI** | **Effectiveness ratio %** |
| --- | --- | --- | --- | --- | --- |
| **Total effect** |  |  |  |  |  |
| X → Y | 0.185 | 0.026 | 0.135 | 0.236 | 100.00 |
| **Direct effect** |  |  |  |  |  |
| X → Y | 0.091 | 0.027 | 0.039 | 0.143 | 49.19 |
| **Total indirect effect** | 0.094 | 0.012 | 0.070 | 0.120 | 50.81 |
| X → M1 → Y | 0.047 | 0.007 | 0.034 | 0.063 | 25.41 |
| X → M2 → Y | 0.038 | 0.009 | 0.023 | 0.056 | 20.54 |
| X → M1 → M2 → Y | 0.009 | 0.002 | 0.005 | 0.014 | 4.86 |

Notes: B, unstandardised coefficient; SE, standard error; Boot LLCI, bootstrap lower limit confidence interval; Boot ULCI, bootstrap upper limit confidence interval. X = Frailty; Y = Multimorbidity; M1 = Sleep quality; M2 = Anxiety. The model was adjusted for age, sex, education level, marital status, annual household income, smoking status, alcohol consumption, dietary habits, and social support.

**Reference**

1. Radloff LS. **The CES-D Scale**. *Appl Psychol Meas.* 2016;**1**(3):385-401. <https://doi.org/10.1177/014662167700100306>.
